# Surgical smoke and ultrafine particles

**DOI:** 10.1186/1745-6673-3-31

**Published:** 2008-12-03

**Authors:** Irene Brüske-Hohlfeld, Gerhard Preissler, Karl-Walter Jauch, Mike Pitz, Dennis Nowak, Annette Peters, H-Erich Wichmann

**Affiliations:** 1 Helmholtz Zentrum München, German Research Center for Environmental Health, Institute of Epidemiology, Neuherberg, Germany; 2 Ludwig-Maximilians-University, Klinikum Grosshadern, Department of Surgery, München, Germany; 3 University of Augsburg, Center for Science and Environment, Augsburg, Germany; 4 Ludwig-Maximilians-University, Institute and Outpatient Clinic for Occupational, Social and Environmental Medicine, München, Germany; 5 IBE (Chair of Epidemiology, Ludwig-Maximilians-University) München, Germany

## Abstract

**Background:**

Electrocautery, laser tissue ablation, and ultrasonic scalpel tissue dissection all generate a 'surgical smoke' containing ultrafine (<100 nm) and accumulation mode particles (< 1 μm). Epidemiological and toxicological studies have shown that exposure to particulate air pollution is associated with adverse cardiovascular and respiratory health effects.

**Methods:**

To measure the amount of generated particulates in 'surgical smoke' during different surgical procedures and to quantify the particle number concentration for operation room personnel a condensation particle counter (CPC, model 3007, TSI Inc.) was applied.

**Results:**

Electro-cauterization and argon plasma tissue coagulation induced the production of very high number concentration (> 100000 cm^-3^) of particles in the diameter range of 10 nm to 1 μm. The peak concentration was confined to the immediate local surrounding of the production side. In the presence of a very efficient air conditioning system the increment and decrement of ultrafine particle occurrence was a matter of seconds, with accumulation of lower particle number concentrations in the operation room for only a few minutes.

**Conclusion:**

Our investigation showed a short term very high exposure to ultrafine particles for surgeons and close assisting operating personnel – alternating with longer periods of low exposure.

## Background

Since the 1990s epidemiological studies from the US and Europe have shown that not only during smog episodes [1], but even at times of relatively low ambient particulate air pollution an increased morbidity and mortality from cardiovascular and pulmonary diseases is observed. There are different studies – including prospective cohort studies [2,3] – reporting associations between daily changes in particulate air pollution and daily mortality [4,5]. An excellent overview is given in the review by Pope and Dockery [6].

Airborne particles are classified according to their aerodynamic diameter: Particles with a diameter smaller than 10 μm (PM_10_) are inhalable and the coarse fraction – between 2.5 and 10 μm – will deposit in the respiratory tract. It will be cleared from it via mucociliary clearance. Insoluble fine particulate matter with a diameter smaller than 2.5 μm (PM_2.5_) precipitates in the alveolar region of the lung, where the only clearance mechanism consists of phagocytosis by alveolar macrophages. PM_2.5 _may induce inflammatory and pro-thrombotic responses, promoting atherosclerosis and thrombogenesis [7]. All these factors contribute to the etiology of cardiovascular disease [8].

PM_2.5 _mass depends mainly on the particle size fraction > 1 μm, whereas particle number concentration is dominated by ultrafine particles with a diameter below 100 nm. The small size facilitates the uptake into cells and transcytosis across epithelial and endothelial cells into the blood and lymph circulation to reach potentially sensitive target sites [9,10]. The greater surface area per unit mass compared with larger-sized particles of the same chemistry renders ultrafine particles biologically more active [11]. For example, interaction between technically produced nanosized particles and mitochondria [12], ion channels [13], and DNA [14] could be identified. In general, the potential toxicity of ultrafine particles will depend on a number of parameters: size, dose, chemistry, persistence, shape, and surface properties, to name just a few.

Electrocautery, argon plasma tissue coagulation, and ultrasonic scalpel tissue dissection all generate a smoke which is generally called 'surgical smoke' containing particles from combustion and numerous chemicals like hydrocarbons, acrylonitrile, fatty acids, and phenols [15]. Factors that can affect the amount of smoke produced include the form of the surgical procedure and the type and intensity of energy applied. Furthermore, it depends on the tissue involved – with the highest emissions originating from burning of organ parenchyma and fatty tissue, and the lowest for musculature. To our knowledge the continuous size distribution of particles in surgical smoke has not been systematically studied. From different investigations it is known that electrocautery creates the smallest particles with a mean aerodynamic size of 0.07 μm [16], whereas laser tissue coagulation creates larger particles (0.31 μm) [17] and the largest particles are generated by use of an ultrasonic scalpel (0.35–6.5 μm) [18].

As several epidemiological studies have found associations of ambient ultrafine particles with adverse respiratory and cardiovascular effects [19-23], it seems reasonable to assume that surgical smoke is potentially dangerous to both patients and personnel in operation rooms, where exposure levels so far are unknown. Our study aimed at measuring the particle number concentration of ultrafine particles during different surgical procedures.

## Methods

The measurements have been carried out during different surgical procedures and in various operation rooms of the "Klinikum Grosshadern" at the Ludwig-Maximilians-University in Munich. The air conditioning system provides 1700 to 2600 m^3 ^air/hour depending on the operating room size.

We used a condensation particle counter (CPC, model 3007, TSI Inc.) measuring number concentration of particles in the diameter size range of 10 nm to 1 μm and a number concentration range between 0 and 100000 particles per cm^3^. Higher concentrations were measured, but cannot be considered as reliable. We tried to standardize the measurement setting by always placing the particle counter at the anesthetist side with the suction tube fixed to the middle of the surgical cover sticking up just about 5 cm corresponding roughly to the breathing zone of surgical personnel.

Particle number concentration (NC) was sampled every second and averaged for a time interval of 5 seconds. The instrument counts particles after they have grown in size in an atmosphere saturated with 2-propanol and formed droplets which can be easily counted by a photodetector as they pass through a laser beam. To test the operational capability of the used CPC 3007 we also performed zero checks by measuring filtered air before each field application.

## Results

**Figure 1:**
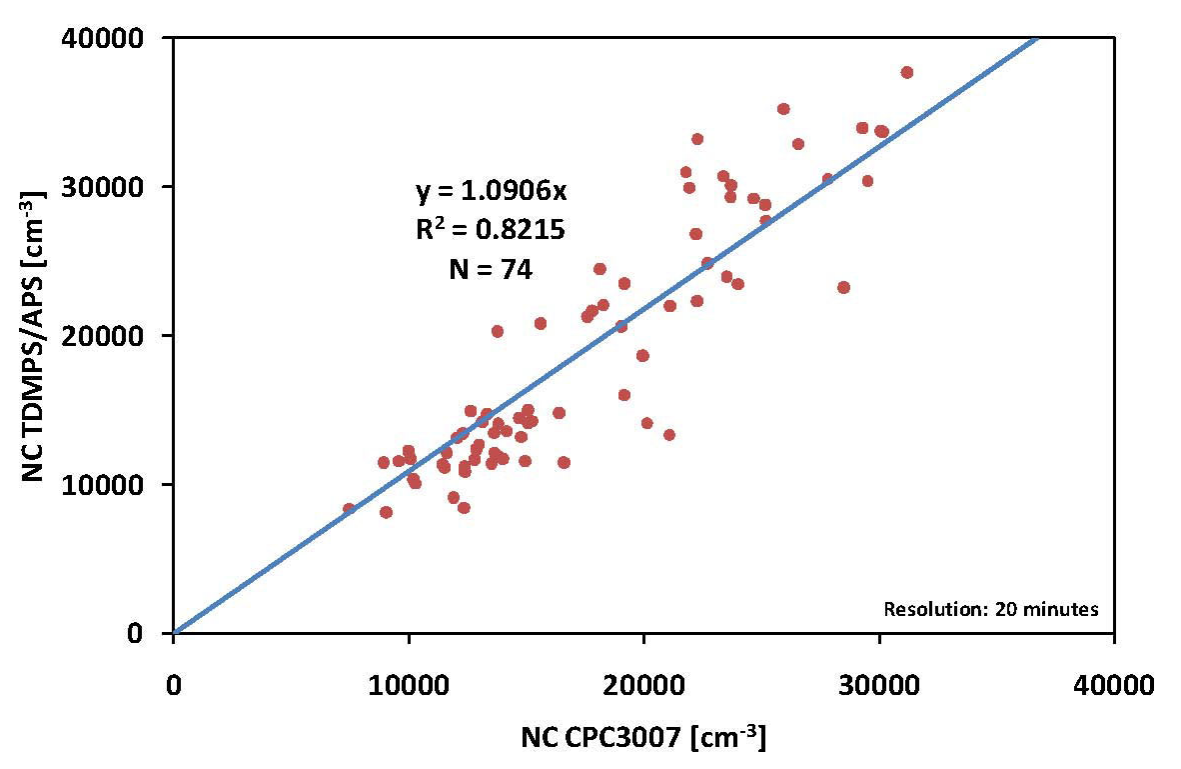
Comparison of particle number concentration measured with the CPC3007 and calculated from the particle size distribution of a TDMPS/APS system.

To validate the measurements of number concentration by the CPC 3007 before performing the study, we used a Twin Differential Mobility Particle Sizer (TDMPS) in combination with a Aerodynamic Particle Sizer (APS), which measured the particle size distribution (PSD) in the diameter range from 3 nm to 10 μm. From the PSD we calculated the NC of particles with diameters of 10 nm to 1 μm and compared these data with the data of the used CPC 3007 (figure 1).

**Figure 2:**
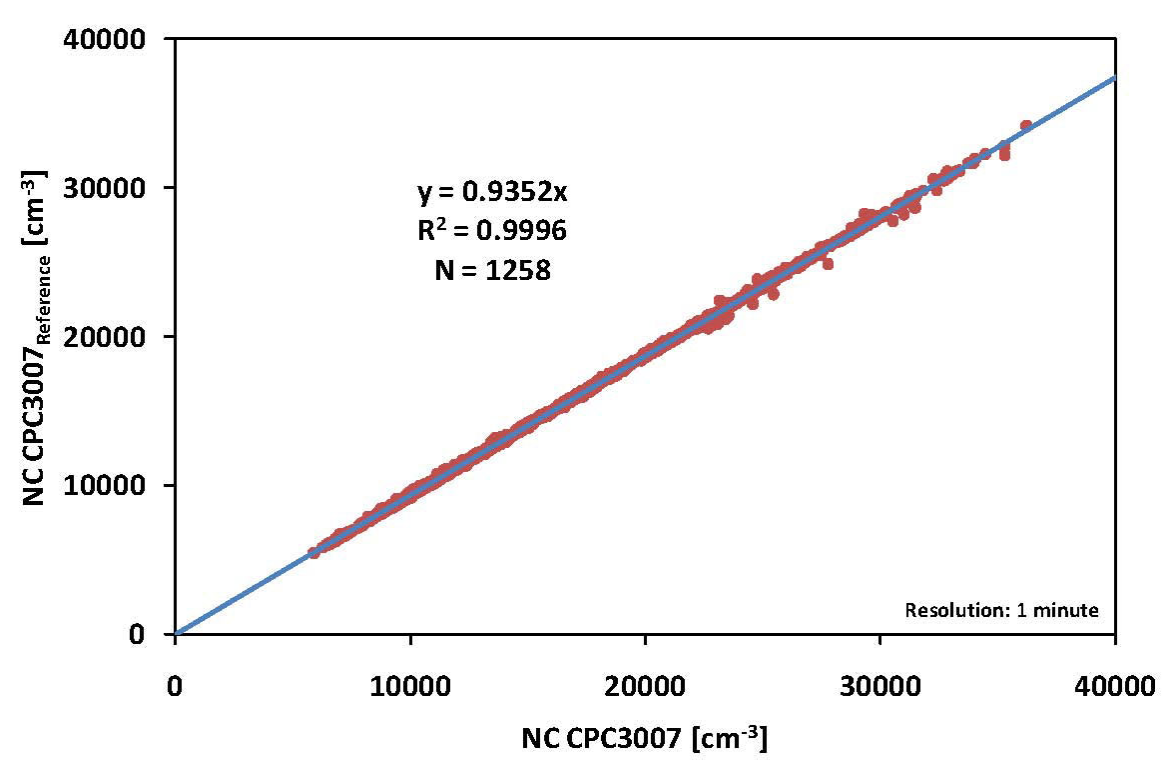
Comparison of particle number concentration measured with two identical CPC3007.

The comparison of the CPC 3007 and the TDMPS/APS in our aerosol laboratory showed good correlation between the two independent measuring methods in total counts of particles (R2 = 0.82) during the test. The observed differences of ~9% clearly underlie the maximum allowable differences of 30% between the moments of the merged PSD and independently measured corresponding parameters [24]. A comparison of the used CPC 3007 with an identical CPC 3007 as a reference (see figure 2) showed very high correlation (variation <7%, R2 = 0.99).

Only such surgical procedures were selected that were expected to be associated with high emissions and only one measurement was carried out for each type of surgical procedure. A summary of the results is presented in table 1.

**Table 1:** Disease, surgical procedure and measurements of ultrafine particle number concentration

Disease	Surgical procedure	Meas. duration	Range min – max	Mean	Standard deviation
		[minutes]	[number per cm^3 ^air]		

local relapse of a retroperitoneal sarcoma	adhesiolysis, tumor removal	169	2 – 183 000	1930	7 970

hemangioma of the liver	hemihepatectomy	193	5 – 490 000	12200	43 100

retroperitoneal tumor	adhesiolysis, tumor removal	73	8 – 32 500	3320	4 840

Incisional hernia	mesh hernia repair	130	13 – 292 000	7210	20 700

benign bile duct stenosis	biliodigestive anastomosis	179	10 – 48 000	1260	3 540

chronic appendicitis	laparoscopic appendectomy	88	5 – 379	74	99

**Figure 3:**
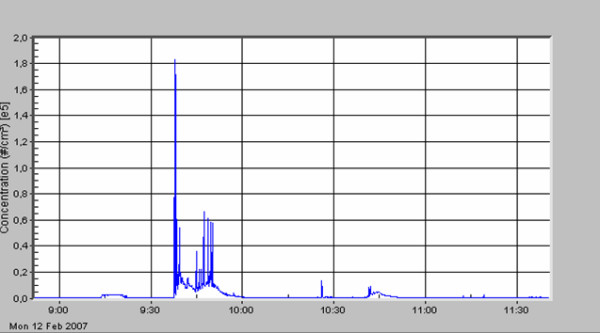
Local relapse of a retroperitoneal sarcoma; adhesiolysis and removal of the tumor.

**Figure 4:**
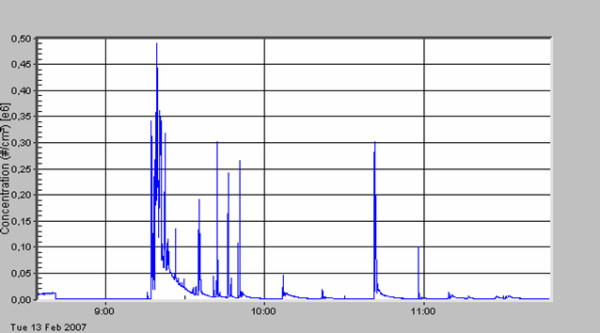
Hemangioma of the liver; hemihepatectomy.

**Figure 5:**
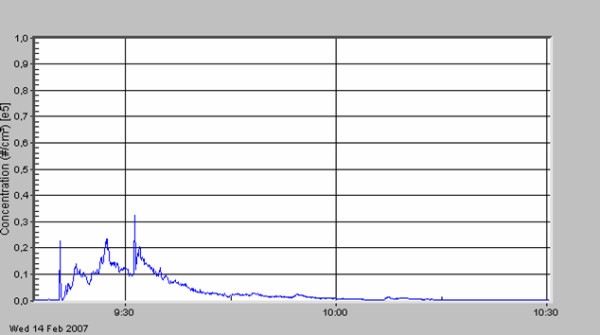
Retroperitoneal tumor; removal of the tumor.

**Figure 6:**
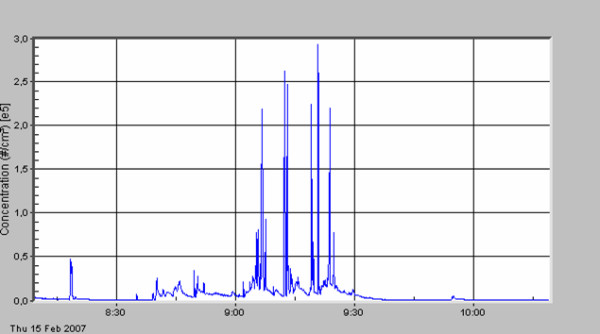
Incisional hernia; mesh hernia repair.

**Figure 7:**
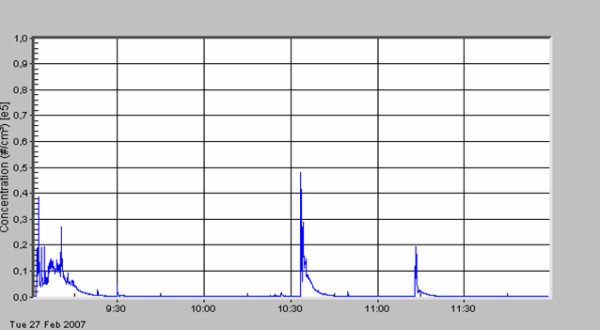
Benign bile duct stenosis; adhesiolysis, biliodigestive anastomosis.

**Figure 8:**
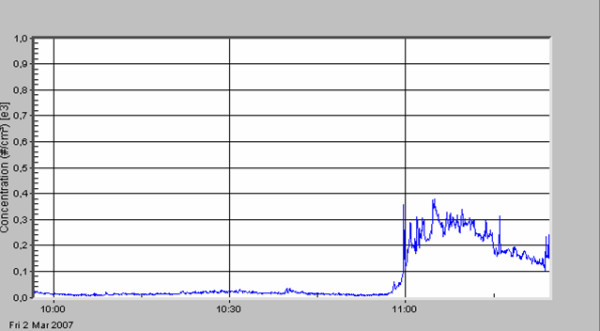
Chronic appendicitis; laparoscopic appendectomy.

The standard deviation describes the very high variation of particle concentration within one surgical procedure. A first peak in particle number concentration was always seen (see figures 3, 4, 5, 6, 7, 8) when performing laparatomy and using electrocauterization for hemostasis of subcutaneous blood vessels. During surgery an abrupt rise in particle concentration could be observed, whenever electro-cauterization or argon plasma tissue coagulation occurred.

When comparing the specific measurements in detail, please note the varying scale of the y-axis in the figures. The mean particle concentration during surgery of a local relapse of a retroperitoneal sarcoma (see figure 3) was 1930 cm^-3^, with a maximum of 183000 cm^-3^, when resolving adhesions by electrocautery. The highest concentrations were found during surgery of a hemangioma of the liver (see figure 4) with a mean of 12200 cm^-3 ^and a maximum of 490000 cm^-3^. The removal of a retroperitoneal tumor was associated with a mean of 3320 cm^-3 ^and a maximum of 32500 cm^-3 ^(see figure 5). Incisional hernia repair was associated with a mean particle exposure of 3320 cm^-3 ^and a peak value of 292000 cm^-3^, while preparing the mesh implantation site between fascia and abdominal musculature using electrocautery (see figure 6). During surgery of a biliary tract stenosis (see figure 7) particle concentration was low with a mean of only 1260 cm^-3 ^and a maximum of 48000 cm^-3 ^during adhesiolysis. Furthermore, using a filter system during laparoscopic appendectomy reduced the mean and the peak particle exposure drastically to values of 74 and 379 cm^-3^, respectively (see figure 8).

The background particle pollution in the operation room in 'Grosshadern' before, during and after surgical procedures was usually below 100 cm^-3^, which is far below the usual concentration inside homes/offices or in the park outside the clinic (~5000 cm^-3^). One operation cubicle specialized for highly sterile orthopedic surgery – containing a laminar air flow chamber – had actually zero particle background contamination, when not operating.

Measuring ultrafine particles in the operation theatre during surgery showed that electro-cauterization and argon plasma tissue coagulation induce the production of very high number concentration of particles in the diameter range of 10 nm to 1 μm. The peak concentration was confined to the immediate local surrounding of the production side. The increment and decrement of ultrafine particle occurrence was a matter of seconds, with accumulation of lower particle number concentrations (< 10000 cm^-3^) in the operation theatre for only a few minutes.

## Discussion

The peaks of very high particle number concentration related to electro-cauterization and argon plasma tissue coagulation were reproducible. But when comparing the results for different surgical procedures quantitatively one should keep in mind that the maximum particle concentrations were outside the measurement range of the CPC equipment and also varied according to the side where the suction tube was fixed in relation to the electrocautery knife or laser emission source. As measurements were only conducted once for every surgical procedure it is impossible to tell to what extent the emissions were "typical" for the procedure.

Our investigation showed a very high exposure to ultrafine particles (> 100000 cm^-3^) for surgeons and close assisting operating personnel – alternating with longer periods of low exposure. Although peaks of ultrafine particles will occur in the majority of cases for only short time intervals, they will accumulate during a professional life. Nothing is known yet about the health effects of such very high and very short (seconds to minutes) exposures to ultrafine particles, but it should be kept in mind that without adequate ventilation the particles produced by electrocautery and laser techniques will probably not vanish quite as quickly.

Ultrafine particles in the surgical smoke have to be looked upon with caution, as they may also contain viable cellular material. For example, intact strands of human papillomavirus DNA have been isolated from carbon dioxide laser plume during treatment of plantar warts [25]. A case report of a laser surgeon who presented with laryngeal papillomatosis was published in 1991 [26]. As the surgeon had given laser therapy to patients with anogenital condylomas, the finding strongly suggested that virus particles present in the laser plume had been transmitted via inhalation.

In general, patients will be protected from particulate air pollution by ventilation with their own supply of oxygen and/or anaesthetic gas. Nevertheless, they can be exposed to high levels of carbon monoxide and hydrogen cyanide during laparoscopic procedures in which smoke is trapped and concentrated in the peritoneal cavity [27,28]. The gases can readily be absorbed from the peritoneum into the bloodstream and synergistically they may impair tissue oxygenation.

Probably the most important safety measure in an operation theatre is a reliable air conditioning system that effectively filters out gases and all freshly produced particles. In addition, local evacuation systems have proven to be highly effective in rapidly eliminating essentially all electrocautery knife smoke and odor [29]. Typically, these filters are comprised of HEPA (***H****igh ****E****fficiency-****P****articulate ****A****rrest*) filters. They are classified upon their efficiency at filtering particulates and their maximum 'inward leakage'. This type of air filter can remove at least 99.97% of airborne particles 0.3 μm in diameter, the size which is considered to be the most penetrating particle size. Particles larger and smaller than 0.3 μm in diameter will be retained more effectively. Infectious virus material as part of the surgical smoke poses a well-documented hazard for operation room personnel that should be taken seriously. Surgical masks do not provide adequate protection. If prolonged periods of electrocautery, laser tissue coagulation, or ultrasonic scalpel use are anticipated, or the potential for transmission of infectious material exists, HEPA filter respirators are preferable to surgical masks.

During endoscopic surgery smoke is accumulated and released at once in a relatively high velocity air flow. Surgeons should pay attention that the jet is not pointed at persons standing close. The preferred preventive measure would be to use a commercially available filter that can be attached to the Luer lock valve on the cannula and that removes cells, particulates, and chemical gases when desufflating.

## Conclusion

Our investigation showed a short term very high exposure to ultrafine particles for surgeons and close assisting operating personnel – alternating with longer periods of low exposure.

## Abbreviations

PM: particulate matter; APS: Aerodynamic Particle Sizer; CPC: Condensation particle counter; TSI: TSI Incorporated, 500 Cardigan Road, Shoreview, MN 55126 U.S.A.; TDMPS: Twin Differential Mobility Particle Sizer

## Competing interests

The authors declare that they have no competing interests.

## Authors' contributions

IBH developed the conception and study design, conducted the measurements and drafted the manuscript. GP and KWJ made substantial contributions to the analysis and interpretation of data. MP was responsible for the validity of measurement data. DN, HEW, and AP have been involved in revising the manuscript. All authors read and approved the final manuscript.
